# Molecular Pathology and Pharmacological Treatment of Autism Spectrum Disorder-Like Phenotypes Using Rodent Models

**DOI:** 10.3389/fncel.2018.00422

**Published:** 2018-11-20

**Authors:** Hsiao-Ying Kuo, Fu-Chin Liu

**Affiliations:** ^1^Institute of Neuroscience, National Yang-Ming University, Taipei, Taiwan; ^2^Brain Research Center, National Yang-Ming University, Taipei, Taiwan

**Keywords:** autism, valproic acid, excitatory/inhibitory imbalance, endocannabinoid system, mTOR signaling, Wnt signaling, neuroinflammation, epigenetics

## Abstract

Autism spectrum disorder (ASD) is a heterogeneous neurodevelopmental disorder with a high prevalence rate. The core symptoms of ASD patients are impaired social communication and repetitive behavior. Genetic and environmental factors contribute to pathophysiology of ASD. Regarding environmental risk factors, it is known that valproic acid (VPA) exposure during pregnancy increases the chance of ASD among offspring. Over a decade of animal model studies have shown that maternal treatment with VPA in rodents recapitulates ASD-like pathophysiology at a molecular, cellular and behavioral level. Here, we review the prevailing theories of ASD pathogenesis, including excitatory/inhibitory imbalance, neurotransmitter dysfunction, dysfunction of mTOR and endocannabinoid signaling pathways, neuroinflammation and epigenetic alterations that have been associated with ASD. We also describe the evidence linking neuropathological changes to ASD-like behavioral abnormalities in maternal VPA-treated rodents. In addition to obtaining an understanding of the neuropathological mechanisms, the VPA-induced ASD-like animal models also serve as a good platform for testing pharmacological reagents that might be use treating ASD. We therefore have summarized the various pharmacological studies that have targeted the classical neurotransmitter systems, the endocannabinoids, the Wnt signal pathway and neuroinflammation. These approaches have been shown to often be able to ameliorate the ASD-like phenotypes induced by maternal VPA treatments.

## Introduction

Autism spectrum disorder (ASD) is a heterogeneous neurodevelopmental disorder with a high prevalence approximately 16.8 in 1,000; these individuals share the core symptoms of impaired social communication/interaction and restrictive/repetitive behavior (American Psychiatric Association, 2013; Baio et al., 2018). In addition to the core symptoms, ASD patients also show various physiological and psychiatric comorbid symptoms, including anxiety, intellectual disability, epilepsy, hypersensitivity, aggression, sleep disturbance, and gastrointestinal problems (Lai et al., 2014). Despite the high prevalence and significant social-economic burdens that ASD imposes, effective treatment(s) are not yet available. There are so far only two U.S. Food and Drug Administration (FDA)-approved drugs; namely risperidone and aripiprazole, that are available for treating irritability in ASD, but none that target the defective social communication (U.S. Food and Drug Administration, 2007, 2009).

Although ASD is a highly heritable disorder (Abrahams and Geschwind, 2008), environmental influences also play a significant role in the etiology of ASD. Risk factors have effects during critical periods in embryogenesis that may enhance susceptibility to ASD. Epidemiological studies have suggested that maternal infection, ethanol exposure and anti-epileptic drug treatment increase the risk of ASD in offspring (Arndt et al., 2005).

A prospective report including 632 subjects has shown that children exposed to valproic acid (VPA) during pregnancy have a significantly higher chance of developing ASD (Bromley et al., 2008). VPA is clinically prescribed as an anti-epileptic and mood-stabilizing drug. VPA is also considered to be a first-line prophylactic drug for migraine headaches. The chemical structure of VPA consists of a simple branched-chain fatty acid (2-propylpentanoic acid) that can indirectly inhibit the enzyme γ-aminobutyric acid (GABA) transferase; this leads to an increase in GABA levels in the brain. It is also important to note that VPA is also able to inhibit voltage-gated Na^+^ channels and this directly suppresses the high-frequency firing of neurons (Rosenberg, 2007). Interestingly, VPA is also known as a non-specific histone deacetylase (HDAC) inhibitor and has been shown to promote the differentiation of carcinoma cells (Földy et al., 2001), neural stem cells (Hao et al., 2004) and adult neural stem cells in the subgranular zone of the hippocampus (Yu et al., 2009). Therefore, VPA has dual pharmacological impacts on VPA-exposed offspring; one effect is reducing the excitability of neural circuits by inhibiting synaptic GABA uptake, the other is epigenetic regulation of chromosome remodeling by inhibiting HDAC activity.

Maternal administration of VPA during pregnancy in rodents results in lifelong abnormalities that recapitulate ASD-like clinical phenotypes. After VPA injection into pregnant rats at embryonic day (E) 12.5 or E13, their offspring exhibit delayed developmental milestones, stereotypic and self-injurious behaviors, and impaired social behavior (Schneider and Przewlocki, 2005; Wagner et al., 2006). These VPA-induced behavioral phenotypes are similar to the major symptoms found in human ASD patients. Subsequent studies have identified abnormal cellular and molecular changes resulting from maternal VPA exposure (Roullet et al., 2013; Nicolini and Fahnestock, 2018). Essentially, the validity of the VPA-induced ASD-like animal model has been discussed widely and it would seem that the biological changes in VPA-treated animals are similar to hypotheses suggested as the origins of ASD; furthermore, the pathological changes and therapeutic response in VPA-treated animals and human patients are similar (Mabunga et al., 2015; Nicolini and Fahnestock, 2018). An advantage of the VPA model is that the ASD-like phenotypes characterized in VPA animals resemble the clinical symptoms of ASD patients. Moreover, the VPA model provides an entry point for researchers when investigating how environmental risk factors can influence the neurodevelopmental processes underlying ASD pathogenesis. It should be noted that VPA exposure during prenatal critical time windows reliably recapitulates the human clinical findings and produces ASD-like phenotypes in rodents ranging from the behavioral level to the molecular level. The maternal VPA-induced ASD-like animal model therefore provides a paradigm by which the etiology of ASD can be experimentally investigated in order to develop potential treatments for ASD. Here, we review the pathological mechanisms of ASD, as well as exploring pharmacological approaches to relieving ASD-like phenotypes using maternal VPA-induced ASD-like animal models (Table 1).

**Table 1:** Summary of the molecular, cellular and behavioral effects of pharmacological treatments in VPA-induced ASD-like animal models.

**Drugs**	**Pharmacologic property**	**Drug dosage/administration**	**Improvements in molecular and cellular endophenotypes**	**Improvements in ASD-related behavior**	**VPA exposure**	**References**
**EXCITATORY/INHIBITORY BALANCE**						
MK-801	NMDAR antagonist	0.3 mg/kg, 4-week-old, 30 min before the behavioral tests		Sociability and social novelty Increase electroshock seizure threshold Hyperactivity	SD rats, 400 mg/kg, E12	Kim et al., 2014b
Agmatine	NMDAR antagonist	25, 50, or 100 mg/kg, 30 min before each test, P26–P53	Reduced over-activation of pERK in the PFC and hippocampus	Sociability and social novelty Repetitive grooming Hyperactivity Increase electroshock seizure threshold	SD rats, 400 mg/kg, E12	Kim et al., 2017a
Memantine	NMDAR antagonist	30 mg/kg, 4-week-old, 30 min before the behavioral tests		Sociability and social novelty Increase electroshock seizure threshold Hyperactivity	SD rats, 400 mg/kg, E12	Kim et al., 2014b
Memantine	NMDAR antagonist	10 or 20 mg/kg/day, P21–P50	Reduced oxidative stress (GSH, TBARS, catalase) Reduced nitrosative stress (nitrate/nitrite) Reduced inflammation (myeloperoxidase activity) in brain and ileum Reduced calcium Increased 5-HT in PFC and ileum Increased activity of mitochondrial enzyme complex-I, II, V in the PFC Restored gastrointestinal tract motility Reduced BBB permeability	Hyperactivity Repetitive behaviors Anxiety Exploratory behaviors Sociability and social novelty	Wistar rats, 500 mg/kg, E12.5	Kumar and Sharma, 2016a
Memantine	NMDAR antagonist	10 mg/kg, 30 min before test, 8- to16-week-old		Sociability Repetitive self-grooming Anxiety	C57BL/6J mice, 600 mg/kg, E13.5	Kang and Kim, 2015
CP465022	AMPAR antagonist	0.25, 0.5, or 1 mg/kg, 30 min before the behavioral tests		Sociability and social novelty Juvenile play	ICR mice, 300 mg/kg, E10	Kim et al., 2018
D-cycloserine	Partial agonist at the glycine binding site on the NMDAR	32 or 64 mg/kg, single injection before tests or daily injection 4 days before the behavioral tests		Vocal communication Social interaction	SD rats, 200 mg/kg, E12, 12.5 and 13	Wellmann et al., 2014
D-cycloserine	Partial agonist at the glycine binding site on the NMDAR	20 mg/kg/day, 4-week-old, 7 days before tests 10 μg/side, infused bilaterally into the lateral amygdala	Enhanced the amplitude of NMDAR-mediated EPSCs Reversed the impaired NMDAR-dependent LTD Reversed the increased spine density in the amygdala Reversed the increased amplitude and frequency of mEPSC in the amygdala Reversed the decreased paired-pulse facilitation ratio Enhanced synaptic removal of GluA2/AMPARs during LTD	Sociability Repetitive behaviors Anxiety	SD rats, 500 mg/kg, E12.5	Wu et al., 2018
MPEP	mGluR5 antagonist	30 mg/kg, 4-week-old, 30 min before the behavioral tests		Social novelty Increase electroshock seizure threshold Hyperactivity	SD rats, 400 mg/kg, E12	Kim et al., 2014b
MPEP	mGluR5 antagonist	30 mg/kg, 4-week-old, 30 min before the behavioral tests		Repetitive self-grooming Anxiety	C57BL/6 mice, 600 mg/kg, E13.5	Kang and Kim, 2015
MPEP	mGluR5 antagonist	20 mg/kg, 7- or 8-week-old, one injection before the behavioral tests		Repetitive self-grooming Compulsive behavior Anxiety	C57BL/6 mice, 600 mg/kg, E13	Mehta et al., 2011
**mTOR PATHWAY**						
Rapamycin	mTOR inhibitor	1 mg/kg/day, P23–P33	Reduced phospho-mTOR, phospho-S6 in the cerebellum, PFC and hippocampus Increased autophagosome, LC3-II, beclin1, atg5 and atg10	Sociability and social novelty Hyperactivity Repetitive behavior	SD rats, 400 mg/kg, E12.5	Qin et al., 2016
Rapamycin	mTOR inhibitor	4 mg/kg/day, P24–P33	Reduced hippocampal apoptosis (*in vitro*) Increased hippocampal Bcl-1 and BDNF	Social interaction Learning and memory	Wistar rats, 600 mg/kg, E12.5	Zhang et al., 2017a
**MONOAMINE**						
8-OH-DPAT	5-HT_1A_ receptor agonist	0.5 mg/kg/day, 7 days before the behavioral tests	Restored the duration and frequency of mEPSC of neurons in the lateral amygdala Restored paired-pulse facilitation in neurons of the lateral amygdala	Social interaction Improve fear memory extinction	SD rats, 500 mg/kg, E12.5	Wang et al., 2013
Melatonin	a pineal hormone synthesized from serotonin	1 or 5 mg/kg/day, P22–40	Restored reduced CaMKII (Thr286), NMDAR1 (Ser896), and PKA (Thr197) phosphorylation in the hippocampus Restored hippocampal LTP	Sociability and social novelty	SD rats, 600 mg/kg, E12.5	Tian et al., 2014
Agomelatine	a dual agonist of melatonin MT1 and MT2 receptors	2 or 4 mg/kg/day, P21–P50	Restored gastrointestinal tract motility Reduced BBB permeability Reduced oxidative stress (GSH, TBARS, catalase) Reduced nitrosative stress (nitrate/nitrite) Reduced inflammation (myeloperoxidase activity) in brain and ileum Reduced calcium Increased 5-HT in the PFC and ileum Increased activity of mitochondrial enzyme complex-I, II, V in the PFC	Sociability and social novelty Repetitive behavior Hyperlocomotion Anxiety Reduced exploratory behavior	Wistar rats, 500 mg/kg, E12.5	Kumar et al., 2015
Ciproxifan	H3R antagonist	3 mg/kg, 30 min before the behavioral tests		Sociability Repetitive behavior	Swiss mice, 500 mg/kg, E11	Baronio et al., 2015
1-(3-(4-tert-pentylphenoxy) propyl)piperidine	H3R antagonist	5, 10, or 15 mg/kg/day, P44–P64, start one week before behavioral tests	Reduced oxidative stress: increase GSH and decrease malondialdehyde Anti-inflammation: reduced pro-inflammatory cytokines IL-1β, IL-6 and TNF-α	Sociability and social novelty Repetitive behavior	Tuck-Ordinary mice, 500 mg/kg, E12.5	Eissa et al., 2018
**ENDOCANNABINOID SIGNALING**						
URB597	Inhibitor of anandamide hydrolysis	2.5 ml/kg, 30 min before the tests at P9 and P13; 2 ml/kg, 2 h before the tests at P35; 1 ml/kg, 2 h before the tests at P90		Events of isolation-induced ultrasonic vocalization Social olfactory discrimination Social play Sociability Repetitive behavior Anxiety	Wistar rats, 500 mg/kg, E12.5	Servadio et al., 2016
PF3845	Inhibitor of fatty acid amide hydrolase	10 mg/kg, 2 h before behavioral test		Sociability	SD rats, 600 mg/kg, E12.5	Kerr et al., 2016
**Wnt SIGNALING**						
Sulindac	NSAID, a β-catenin inhibitor	5 mg/kg after VPA administration	VPA treatment in the cortical primary culture system: *in vitro* Decreased mRNA expression of β-catenin Decreased phosphorylation of GSK-3β Increased phosphorylation of β-catenin Decreased oxidative stress indicated by 4-Hydroxynonenal The tissue of VPA-treated rats: *in vivo* Reversing the increased levels of phospho-GSK3β and 4-hydroxynonenal protein Reversing the reduced phospho-β-catenin	Repetitive rearing and self-groomingHyperactivityReduce nociceptive sensitivityLearning and memory	Wistar rats, 600 mg/kg, E12.5	Zhang et al., 2012, 2015
Sulindac	NSAID, a β-catenin inhibitor	5 mg/kg/day, P23–P33	Restored reduced β-catenin, phospho-GSK3β, phospho-mTOR Increased LC3-II	Sociability and social novelty Hyperactivity Repetitive behavior	SD rats, 400 mg/kg, E12.5	Qin et al., 2016
Wortmannin	PI3K inhibitor for suppression of phosphorylation of GSK-3β and β-catenin	2.5 μg/site, intra-amygdaloid infusion, 1 week before the behavioral tests	Reduced phospho-GSK3β and β-catenin	Sociability	SD rats, 500 mg/kg, E12.5	Wu et al., 2017b
**ANTIBIOTICS, COMPOUNDS OR FOOD/NUTRITION FOR ANTI-INFLAMMATION**						
Minocycline	Possible inhibitor of microglia activation Tetracycline antibiotic which is used in psychiatry for its pleiotropic anti-inflammatory and neuroprotective effects	25 or 50 mg/kg/day, P21–P50	Restored gastrointestinal tract motility Reduced BBB permeability Reduced oxidative stress (GSH, TBARS, catalase) Reduced nitrosative stress (nitrate/nitrite) Reduced inflammation (myeloperoxidase activity) in brain and ileum Reduced calcium Increased 5-HT in the PFC and ileum Increased activity of mitochondrial enzyme complex-I, II, V in the PFC	Repetitive behavior Hyperactivity Sociability and social novelty Anxiety Increase exploratory behavior	Wistar rat, 500 mg/kg, E12.5	Kumar and Sharma, 2016b
Resveratrol	Polyphenol compound with anti-oxidative, anti-inflammatory and neuroprotective properties	3.6 mg/kg, E6.5–E18.5	Restored abnormal distribution of pyramidal neurons and parvalbumin-positive GABAergic interneurons in the primary somatosensory cortex Restored density of parvalbumin neurons in the amygdala Restored gephyrin expression in the primary somatosensory cortex	Sociability and social novelty Sensorial behaviors including nest seeking and whisker nuisance task	Wistar rat, 600 mg/kg, E12.5	Bambini-Junior et al., 2014; Fontes-Dutra et al., 2018
Astaxanthin	Antioxidant and neuroprotectant	2 mg/kg, P26–P56	In brain, reduced level of lipid peroxidation, nitric oxide and advanced protein oxidation product; increased catalase activity, and superoxide dismutase activity and GSH In liver, a reduced level of advanced protein oxidation product, nitric oxide, catalase activity, and superoxide dismutase activity	Reduce reaction time in hot plate test Sociability and social novelty Hyperactivity	Swiss albino mice, 600 mg/kg, E12.5	Al-Amin et al., 2015
Piperine	Antioxidant Neuroprotectant Reduce serotonin level	5 or 20 mg/kg/day, P13–P40	Oxidative stress: reduced malondialdehyde and nitrite; increased GSH Reduced hippocampal serotonin Restored integrity of Purkinje cell layer in the cerebellum	Social behaviorAnxietyHyper-locomotionNegative geotaxisNociceptive responseMotor activity on rotarodLearning and memory	BALB/c mice, 400 mg/kg, P14	Pragnya et al., 2014
DHA	Component of the phospholipid structure of cellular membranes in the brain	75, 150, or 300 mg/kg/day, P14–P35	Increased DHA in the hippocampus and plasma Reduced neuronal damage in CA1 and CA3 Reduced cell apoptosis and activity of caspase 3 Restored p-Akt, Bcl-2, Bax, caspase 3, p-CaMKII, p-CREB Reduced peroxidation	Learning and memory	Wistar rat, 600 mg/kg, E12.5	Gao et al., 2016
Green tea extract	Antioxidant, quencher of free radicals as it diminishes the generation of lipid peroxides by polyphenols that are capable of crossing BBB	75 or 300 mg/kg/day, P13–P40	Reduced lipid peroxidation Reversing the cell loss of Purkinje cell layer of the cerebellum	Negative geotaxis Mid-air righting Thermal nociception Hyperactivity Motor activity Exploratory activity Anxiety Learning and memory	Albino mice, 400 mg/kg, P14	Banji et al., 2011
Korean red ginseng extract	Increase cerebral blood flow, and inhibit superoxide production	20, 50, 100, and 200 mg/kg/day, E10–E15	Reduced crooked tail phenotype (neural tube defect)	Sociability and social novelty Hyperactivity Electroshock seizure threshold	SD rats, 400 mg/kg, E12	Kim et al., 2013
Korean red ginseng extract	Increase cerebral blood flow, and inhibit the superoxide production	100 or 200 mg/kg/day, P21–P38		Sociability and social novelty Hyperactivity Repetitive behavior Electroshock seizure threshold	ICR mice, 300 mg/kg, E10	Gonzales et al., 2016
Combined extract of purple rice and silkworm pupae	Antioxidant that contained 2,2-diphenylpicrylhydrazyl and phenol	50, 100, and 200 mg/kg/day, P14–P40	Reduced the oxidative stress in the cerebellum: increase catalase, superoxide dismutase, GSH; decrease malondialdehyde Increased cell size and density of Purkinje cells of the cerebellum	Social behavior Anxiety Negative geotaxis Mid-air righting reflex Learning and memory Sensorimotor coordination	Rat, 400 mg/kg, P14	Morakotsriwan et al., 2016
Co-ultraPEA-LUT	Palmitoylethanolamide (PEA): an endogenous fatty acid amide; ligand of peroxisome proliferator-activated receptor alpha; enhance anandamide activity; anti-inflammatory, anti-nociceptive, neuroprotec-tive, and anticonvulsant properties Luteolin (LUT): flavones act as neuroprotectant	1 mg/kg/day, P15–P30 or 3 months from P15	Anti-inflammation: reduced iNOS, GFAP and NF-κB in the hippocampus and cerebellum; reduced TNF-alpha, IL-1b, chymase and tryptase in the hippocampus; increased IκB-alpha Anti-apoptosis with reduced Bax and increased Bcl-2 in the hippocampus and cerebellum Increased adult neurogenesis Increased hippocampal spine density	Sociability Anxiety	C57BL/6 mice, 400 mg/kg, P14	Bertolino et al., 2017
Vitamin D3	Neuroprotectant	80,000 IU/kg, P12	Reversing the decreased 25(OH)D3 at P45	Repetitive grooming Social interaction Weight gain during P7-P12 Eye-opening Swimming coordination Olfactory capacity	Wistar rats, 600 mg/kg, E12.5	Du et al., 2017
**HDAC INHIBITORS/EPIGENETIC REGULATORS**						
Pentyl-4-yn-VPA	HDAC inhibitor, analog of VPA	84 mg/kg/day, P72–P90 during behavioral tests	Enhanced cerebellar H4K8 and H3K14 acetylation Decreased cerebellar H3K9 acetylation	Social interaction Spatial learning and memory	Wistar rats, 600 mg/kg, E12.5	Foley et al., 2014
Suberoylanilide hydroxamic acid	Pan-specific HDAC inhibitor	5 mg/kg/day, P72–P90 or P72–P93 during behavioral tests	Enhanced cerebellar H4K8 acetylation Increased polysialylation state of neural cell adhesion molecules in the dentate gyrus	Social interaction Biological motion perception Spatial learning	Wistar rats, 600 mg/kg, E12.5	Foley et al., 2012, 2014
MS-275	HDAC 1-3 inhibitor	1 mg/kg/day, P72–P93 during behavioral tests	Increased polysialylation state of neural cell adhesion molecules in the dentate gyrus	Social interaction Water maze spatial learning	Wistar rats, 600 mg/kg, E12.5	Foley et al., 2012
VPA	HDAC inhibitor	30 mg/kg/day, 5 weeks from 4-week-old	Increased dendritic spines of CA1 neurons in the hippocampus	Long-term recognition memory	ICR mice, 500 mg/kg, E12.5	Takuma et al., 2014
Sodium butyrate	HDAC inhibitor	1.2 g/kg/day, 5 weeks from 4-week-old	Increased dendritic spines of CA1 neurons in the hippocampus Increased the acetylated H3 level of CA1 neurons	Long-term recognition memory	ICR mice, 500 mg/kg, E12.5	Takuma et al., 2014
**ANTIPSYCHOTICS**						
Risperidone	Atypical antipsychotics that inhibits D2R and 5-HT_2A_ receptor	0.2 mg/kg/day, P0–P7	Reversing abnormal striosomal compartmentation in the striatum	Duration of isolation-induced ultrasonic vocalizations	FVB mice, 400 mg/kg, E12.5	Kuo and Liu, 2017
Risperidone	Atypical antipsychotics that inhibits D2R and 5-HT_2A_ receptor	0.2 mg/kg, 24h or daily for 2 weeks before the test	Reversing reduced dendritic spine density in the PFC	Social interaction Recognition memory	ICR mice, 500 mg/kg, E12.5	Hara et al., 2017
Aripiprazole	Atypical antipsychotics that acts as 5-HT_2A_ receptor agonist and partial agonist of D2R	3 mg/kg, 24h or daily for 2 weeks before the test	Reversing reduced dendritic spine density in the PFC	Social interaction Recognition memory	ICR mice, 500 mg/kg, E12.5	Hara et al., 2017
Atomoxetine	Blocker of norepinephrine transporter for treating ADHD	3 mg/kg		Hyperactivity Repetitive rearing	SD rats, 400 mg/kg, E12	Choi et al., 2014
Atomoxetine	Blocker of norepinephrine transporter for treating ADHD	1 mg/kg/day, 2 weeks before the behavioral tests from about 8-week-old	Restored density of dendritic spines in the PFC	Social interaction Recognition memory	ICR mice, 500 mg/kg, E12.5	Hara et al., 2016
Methylphenidate	Blockers of norepinephrine and dopamine transporter for treating ADHD	3 mg/kg/day, 2 weeks before the behavioral tests from about 8-week-old	Increased prefrontal dopamine and noradrenaline release Restored density of dendritic spines in the PFC	Social interaction Recognition memory	ICR mice, 500 mg/kg, E12.5	Hara et al., 2016
**OTHER OFF-LABEL DRUGS**						
Bumetanide	Specific NKCC1 chloride importer antagonist	2–2.5 mg/kg in drinking water one day before delivery	Shifting the driving force of GABA_A_R from depolarizing to hyperpolarizing at P0 Reduced glutamatergic spontaneous EPSC of hippocampal CA3 pyramidal neurons Switching the action of GABA from excitatory to inhibitory at P15 Reduced network oscillation power	Events and duration of isolation-induced ultrasonic vocalization Sociability	Wistar rats, 600 mg/kg, E12.5	Eftekhari et al., 2014; Tyzio et al., 2014
Donepezil	Inhibition of acetylcholine esterase, stimulation of alpha-7 nicotinic acetylcholine receptors, and endocytosis of NMDARs	0.3 mg/kg/day, P14–P40	Reduction of the increased AChE activity in the PFC	Sociability and social preference Nest building Marble burying test Anxiety Novel object recognition	SD rats, 400 mg/kg, E12 ICR, 300 mg/kg, E10	Kim et al., 2014a
N-acetylcysteine	Antioxidant and a precursor of GSH for treating acetaminophen overdose	150 mg/kg/day, 10 days before behavioral test from P21	Increased frequency of mEPSC in the amygdala	Social interaction (juvenile play) Anxiety Improved social interaction by N-acetylcysteine was blocked by mGluR2/3 inhibitor	SD rats, 500 mg/kg, E12.5	Chen et al., 2014
N-acetylcysteine	Antioxidant and a precursor of GSH for treating acetaminophen overdose	150 mg/kg/day, 4 weeks before behavioral tests	Increased GSH Reduced malondialdehyde expression	Repetitive behavior	SD rats, 600 mg/kg, E12.5	Zhang et al., 2017b
Fingolimod	Oral immunosuppressant Treatment of multiple sclerosis; Anti-apoptosis and anti-inflammation	0.25, 0.5, or 1 mg/kg, P15–P35	Increased hippocampal cell/neuronal density Anti-inflammation: decreased the level of S1P and S1Pr1 protein expression; decreased Iba-1, IL-6, IL-1beta in the hippocampus Anti-oxidation: decreased malondialdehyde; increased superoxide dismutase, GSH, GSH-PX Anti-apoptosis: decreased Bax and caspase 3; increased Bcl-2 Increased phospho-CaMKII, p-CREB, BDNF	Sociability and social novelty Learning and memory	Wistar rats, 600 mg/kg, E12.5	Wu et al., 2017a

AChE, acetylcholinesterase; ADHD, attention deficit hyperactivity disorder; BBB, blood-brain barrier; BDNF, brain-derived neurotrophic factor; DHA, docosahexaenoic acid; E, embryonic day; GSH, glutathione; LTD, long-term depression; mEPSC, miniature excitatory postsynaptic currents; NKCC1, Na-K-Cl cotransporter 1; NSAID, non-steroidal anti-inflammatory drug; P, postnatal day; PFC, prefrontal cortex; S1P, sphingosine 1-phosphate.

## The excitatory/inhibitory imbalance hypothesis

An influential theory regarding psychiatric diseases over the last two decades is one that involves an imbalance affecting the excitatory/inhibitory (E/I) neural circuits (Rubenstein and Merzenich, 2003; Marín, 2012; Nelson and Valakh, 2015). Hussman (2001) first proposed that hypofunction of GABAergic neurotransmission as part of ASD pathophysiology (Hussman, 2001). Rubenstein and Merzenich (2003) further postulated that an increased E/I ratio might be a pathogenic mechanism for ASD. The E/I imbalance theory is based on the observation that there are higher incidences of epilepsy and abnormal GABAergic function in patients with ASD. The theory proposes that a loss of inhibitory control may cause elevated noise in the brain's networks and this alters sensory, emotional and social information processing, which in turn reduces the adaptive ability of ASD patients to process and respond to environmental stimulation (Rubenstein and Merzenich, 2003). Supporting evidence has come from clinical and genetic mouse model studies that have shown a disruption of E/I homeostasis may result from defective GABAergic inhibitory input, abnormal glutamatergic transmission and/or homeostatic compensation (Nelson and Valakh, 2015). In ASD models involving maternal VPA treatment, NMDA receptor (NMDAR)-mediated currents are increased in the medial prefrontal cortex (mPFC) in maternal VPA-treated rats before P16, but not between P30 and P50 (Rinaldi et al., 2007; Walcott et al., 2011). It is worth noting that the reduction in NMDAR function during adulthood may reflect a compensatory homeostasis for the presence of an E/I imbalance during development (Martin and Manzoni, 2014). Therefore, an E/I imbalance would seem to be involved in the pathogenesis of ASD-like phenotypes that are induced by VPA.

Based on the E/I imbalance theory, several VPA-induced ASD model studies have tested whether activity-dependent manipulation via glutamate receptors is beneficial and able to alleviate ASD-like phenotypes. Clinical studies have shown that genetic mutation of the GRIA2 and GRIA3 subunits of AMPA receptors (AMPAR), are associated with ASD (Ramanathan et al., 2004; Jacquemont et al., 2006). In VPA model systems, GluA1 protein levels and mEPSC amplitudes are increased in the mPFC of VPA-treated mice; furthermore, AMPA antagonist treatment is able to improve the social deficits present in VPA-exposed mice (Kim et al., 2018). Along with this, *de novo* mutation of *GRIN2B*, a subunit of the NMDAR, has also been found in patients with ASD (Tarabeux et al., 2011; O'Roak et al., 2012a). Consistent with findings observed in human patients, the GRIN2A and GRIN2B subunits of the NMDAR are upregulated in the primary somatosensory cortex of VPA-treated rats (Rinaldi et al., 2007). Interestingly, there is increased local connectivity and NMDAR-mediated long-term potentiation (LTP) but there is also reduced connective strength and excitability in the mPFC and the primary somatosensory cortex of rodents after treatment with VPA in the second postnatal week (Rinaldi et al., 2007, 2008). Based on these findings, NMDAR antagonists have been tested as potential drugs for alleviating the ASD-like phenotypes of VPA-treated rodent offspring. A reduction in the threshold for electronic shock seizure in VPA-treated offspring has been found and this mimics the higher susceptibility of epilepsy in ASD patients. The NMDAR blockers MK-801 and agmatine increase the threshold of electronic shock seizure of VPA-treated rats (Kim et al., 2014b, 2017a). NMDA antagonists, including MK-801, agmatine and memantine, relieve the hyperactivity, anxiety, social impairments and the repetitive behavior of VPA-treated rats (Kim et al., 2014b, 2017a; Kang and Kim, 2015; Kumar and Sharma, 2016a). Moreover, administration of donepezil, an acetylcholine esterase inhibitor that is able to induce endocytosis of the NMDAR, improves VPA-induced ASD-like phenotypes (Kim et al., 2014a). Interestingly, D-cycloserine, a partial NMDA agonist that acts on the glycine binding site of NMDAR, not only decreases NMDA function in the amygdala, but also alleviates anxiety, social and vocal communication in VPA-treated rats (Wellmann et al., 2014; Wu et al., 2018). D-cycloserine treatment also enhances the endocytosis of NMDAR at synaptic sites during long-term depression (LTD) induction in the amygdala of the VPA-treated rats. These findings suggest that D-cycloserine is able to regulate the E/I neuronal activity balance via modulation of NMDA function (Wu et al., 2018).

In addition to ionotropic glutamate receptors, metabotropic glutamate receptors (mGluR) have also been implicated in ASD pathogenesis. For example, hyperfunction and hypofunction of mGluR5 signaling is known to be involved in Fragile X syndrome and tuberous sclerosis complex (TSC), respectively (Auerbach et al., 2011). ASD-like phenotypes induced by the *Fmr1* mutation, a gene responsible for Fragile X syndrome, are able to be alleviated by administration of mGluR antagonists in mouse models (Choi et al., 2011; Thomas et al., 2012). Furthermore, a recent study has shown that there is a higher prevalence of copy number variation of the gene *RANBP1*, which affects the mGluR and mGluR network, in ASD patients (Wenger et al., 2016). In the VPA model system, disruption of the E/I balance is accompanied by reduced levels of mGluR2/3 in the lateral amygdala of VPA-treated mouse offspring (Lin et al., 2013; Chen et al., 2014). Furthermore, a mGluR5 antagonist is capable of relieving ASD-like behaviors, including repetitive behavior and anxiety (Mehta et al., 2011; Kang and Kim, 2015).

ASD is highly heterogeneous and therefore the E/I imbalance observed in ASD brains may be a result of dysfunction of either or both the excitatory and inhibitory synapses, which suggests that there are aberrant developmental processes affecting multiple cell types contributing this whole complex of abnormalities. During early development, a high level of sodium-potassium-chloride cotransporter 1 (NKCC1) results in an increase in intracellular chloride and this then leads to the excitatory effects of GABA. After the end of the first week after birth, GABA's effects become inhibitory due to the gradual expression of chloride exporter potassium-chloride transporter 2 (Rivera et al., 1999; Valeeva et al., 2013). Oxytocin is known to induce a transient reduction in intracellular chloride levels and thus it can cause a temporary GABA switch during fetus delivery (Tyzio et al., 2006). However, this transient reduction in intracellular chloride level is absent in VPA-treated mouse offspring; this effect may be related to aberrant neonatal vocal communication, abnormal social behavior, and neuronal oscillation during adulthood (Eftekhari et al., 2014; Tyzio et al., 2014). These ASD-like phenotypes are able to be rescued by perinatal administration of an antagonist of NKCC1, which augments the transient reduction in intracellular chloride during fetus delivery (Eftekhari et al., 2014; Tyzio et al., 2014). These results not only help to elucidate GABA-mediated network maturation, but also provide evidence to support the hypothesis than an E/I imbalance can be a therapeutic target when treating ASD.

## Abnormal mTOR signaling as part of ASD pathophysiology

The presence of abnormal mammalian target of rapamycin (mTOR) and related mTOR-mediated signaling has been demonstrated in several syndromic ASD models, including TSC, Fragile X syndrome, Rett syndrome, and Angelman syndrome (Winden et al., 2018). mTOR is a core kinase of mTORC1 and mTORC2, which are regulated by Wnt signaling and PI3K pathway. The mTOR signal pathway integrates various cellular signals related to ATP-mediated energy metabolism, trophic factors, neurotransmitters and amino acids (Lipton and Sahin, 2014). Under physiological conditions, mTOR signaling controls cell survival, cell proliferation, the cytoskeleton, mRNA translation, protein synthesis, and autophagy. These mTOR-mediated basic cellular functions are involved in the control of extensive neurodevelopmental processes including neurogenesis, neuronal migration, axonogenesis, synaptogenesis, and circuity formation (Lipton and Sahin, 2014; Crino, 2016; Winden et al., 2018). The genes *TSC*, phosphatase and tensin homolog (*PTEN*) and neurofibromatosis type 1 (*NF1*), which are negatively regulated by mTOR kinase activity, are responsible for syndromic ASD pathogenesis in various diseases including TSC, PTEN hamartoma tumor syndrome, and RASopathies, respectively (Winden et al., 2018). Consistently, over-activation of mTOR signaling has been found in the cerebral cortex of human postmortem ASD brains (Tang et al., 2014). Rapamycin is an antibiotic that binds to the FKPB12-rapamycin binding site of mTOR and inhibits mTORC1, but not mTORC2, activity. Rapamycin was originally used as an immunosuppressant during tissue transplantation. In ASD transgenic mice models, rapamycin has been tested as a potential pharmacotherapic agent to treat ASD. For example, it has been shown that the abnormal social interaction and stereotypic behaviors are ameliorated by rapamycin treatment in heterozygotes carry one copy of the *TSC* knockout and in neuronal, astroglial or cerebellar Purkinje cell-specific conditional *TSC* knockout mice (Meikle et al., 2008; Zeng et al., 2008; Sato et al., 2012; Tsai et al., 2012; Tang et al., 2014). In addition to rescuing behavioral deficits, rapamycin also improves several endophenotypes of the *TSC* mutant models, including abnormal myelination, reduced spinogenesis and blockade of autophagy (Meikle et al., 2008; Sato et al., 2012; Tsai et al., 2012; Tang et al., 2014). In an ASD model in which *Pten* is conditionally knocked out in oligodendrocyte and Schwann cells, rapamycin treatment was found to reduce the hypertrophy of white matter (Goebbels et al., 2010). These findings support the hypothesis that the mTOR pathway is a potential intervention target for ASD.

Abnormal up-regulation of mTOR signaling is observed in the brains of VPA-treated rats (Qin et al., 2016; Zhang et al., 2017a). Increased phospho-mTOR and phospho-S6, a downstream target of mTOR, have been found in the mPFC, hippocampus and cerebellum of VPA-treated brains and these changes were associated with reduced autophagosome and increased apoptosis (Qin et al., 2016; Zhang et al., 2017a). Treating VPA mice with rapamycin not only is able to improve social impairment, hyperactivity, repetitive behavior, and learning/memory, but it also increases BDNF and Bcl-2 expression (Qin et al., 2016; Zhang et al., 2017a). Moreover, the impairment of autophagy is ameliorated by rapamycin treatment as a result of up-regulation of the components of autophagosomes in the cerebellum, mPFC and hippocampus; this was observed after chronic rapamycin treatment of VPA-treated rats (Qin et al., 2016). Taken the above findings together, a potential ASD therapeutic approach involving a reduction in the over-activated mTOR signal transduction is supported by investigations that have used a VPA rodent model.

## Dysregulation of monoamine transmission in ASD

Abnormalities of the various monoaminergic systems, including serotonin, catecholamine and histamine, have been observed in ASD patients (Lake et al., 1977; Gabriele et al., 2014; Wright et al., 2017). VPA-treated animals also exhibit aberrant monoamine transmission (see references below). We discuss here the involvement of monoamines in the pathophysiology of ASD and current progress regarding therapeutic strategies that focus on the monoamines systems.

### Serotonin

Serotonin (5-hydroxytryptamine, 5-HT) is a neurotransmitter known to be involved in regulating psychiatric function. 5-HT is synthesized from tryptophan by a two-step process. Tryptophan is first converted into 5-HTP by tryptophan hydroxylase (TPH). 5-HTP is then converted into 5-HT by aromatic acid decarboxylase (AADC). Serotonin is transported into synaptic vesicles by vesicular monoamine transporter (VMAT). When released, serotonin in the synaptic cleft is taken up by serotonin transporter (SERT) into the presynaptic terminal in order to terminate neurotransmission and to allow it to be recycled (Frazer and Hensler, 1999).

Hyperserotonemia was first detected in early onsets infantile ASD children, but six out of 23 of the ASD children with highest 5-HT levels were reported in this study to not show specifically correlated clinical signs (Schain and Freedman, 1961). Elevated levels of blood 5-HT have been reported by a meta-analysis study to be present in 25.4% of ASD patients (Gabriele et al., 2014). However, neuropathological studies of human ASD postmortem brains are not consistent with clinical studies. Azmitia et al. (2011) reported that the number of SERT-immunoreactive axons is increased in the 5-HT fibers innervating the cortex and forebrain of ASD brains (Azmitia et al., 2011). On the other hand, Oblak et al. (2013) showed that there were significant reductions in SERT, 5-HT receptor 1A (5-HT_1A_ receptor) and 5-HT receptor 2A (5-HT_2A_ receptor) in the posterior cingulate cortex and fusiform gyrus of human ASD postmortem brains; these two cortical regions are involved in social-emotional processing (Oblak et al., 2013). In addition, genetic linkage studies have identified variants of the SERT gene, *SLC6A4*, as being present in autistic patients, and that this was found to be correlated with impaired social communication by the ASD patients (Cook et al., 1997; Tordjman et al., 2001). A later study further indicates that such *SLC6A4* variants are correlated with platelet hyperserotonemia in ASD individuals (Coutinho et al., 2004). Despite the presence of some inconsistencies, a meta-analysis study has shown that prenatally exposure to selective serotonin reuptake inhibitors (SSRI) leads to a higher odds ratio of having a offspring with ASD (Man et al., 2015). Notably, ASD patients with short-term depletion of tryptophan show deteriorated repetitive behavior and a poorer anxiety status (McDougle et al., 1996), which suggests that a dysregulation in serotonin function is a risk factor for ASD (Muller et al., 2016).

Serotonin is a precursor of melatonin, which is produced in the pineal gland and is involved in the regulation of circadian rhythms (Frazer and Hensler, 1999). Melatonin is relevant to ASD because there is a high prevalence of sleep disturbance among ASD patients (Rossignol and Frye, 2011; Tordjman et al., 2013). Abnormal melatonin biosynthesis and reduced levels of melatonin have been found in children and adults who are ASD patients (Nir et al., 1995; Kulman et al., 2000; Melke et al., 2008). Treatment with melatonin is able to reduce such sleep disturbance as well as autistic behavior among ASD individuals (Rossignol and Frye, 2011). It should be noted that sleep problems are also found in patients with other psychiatric diseases, such as schizophrenia, depression and anxiety (Krystal, 2012). The specificity, the mechanisms involved and the causality of the therapeutic effects of melatonin treatment of ASD need to be further clarified in future studies.

### Catecholamine

Dopamine and norepinephrine, two major catecholamines in the brain, are both synthesized from tyrosine. Tyrosine hydroxylase first converts tyrosine into L-DOPA. Subsequent decarboxylation of L-DOPA leads to the production of dopamine. After release from presynaptic sites, dopamine binds to dopamine receptors within the postsynaptic sites. Dopamine can be cleared from synaptic clefts by uptake into the presynaptic terminals via the dopamine transporter (DAT) (Kuhar et al., 1999). Early evidence pointing to catecholamine dysfunction in ASD patients is based on the finding that in ASD patients there are increased plasma levels of norepinephrine and decreased plasma dopamine-β-hydroxylase activity levels, the latter being the enzyme that converts dopamine to norepinephrine (Lake et al., 1977). Genetic mutation of the regulators of dopaminergic neurotransmission have been identified as present in ASD patients (Nguyen et al., 2014), and these include *DRD1* (Hettinger et al., 2008), *DRD2* (Hettinger et al., 2012), *DRD3* (de Krom et al., 2009; Staal et al., 2015), *DRD4* (Gadow et al., 2010), and *DAT* (Hamilton et al., 2013). Notably, the midbrain dopamine systems seem to be involved in the pathology of autism. The dopaminergic mesolimbic and mesocortical pathways have been shown to participate in the reward circuits associated with social motivation and social interaction (Dichter et al., 2012; Gunaydin and Deisseroth, 2014). Reduced dopamine levels in the mPFC of medication-free ASD children, as measured by positron emission tomographic scanning, suggest that hypofunction of the dopamine reward system may be involved in the social communication deficits of ASD patients (Ernst et al., 1997). The dopaminergic mesostriatal pathways have been shown to control movement, and degeneration of the mesostriatal pathways is a hallmark of Parkinson's disease (Iversen and Iversen, 2007). Abnormalities affecting the mesostriatal pathways have been linked to repetitive behavior in ASD. This is based on the fact that drug-induced changes in the mesostriatal pathways result in altered stereotypic behaviors in rats (Iwamoto et al., 1976). Given the importance of the mesostriatal pathways to reward prediction, motivational control, and decision making (Matsumoto and Hikosaka, 2009; Ilango et al., 2014; Lerner et al., 2015), it seems highly likely that these pathological changes in the midbrain dopamine systems may be involved in ASD pathogenesis.

### Histamine

The histaminergic system has been postulated to be involved in the pathophysiology of neurodevelopmental disorders, especially schizophrenia and ASD (Baronio et al., 2014; Wright et al., 2017). Histamine is derived from histidine by decarboxylation and is largely released by the histaminergic neurons present in the tuberomammillary nucleus of the hypothalamus. The extracellular histamine is inactivated and converted into tele-methylhistamine by neuronal histamine N-methyltransferase (HNMT) in the brain (Haas et al., 2008). The histaminergic system is believed to play an important role in regulating a variety of physiological function; these include the sleep/awake cycle, addiction, neuroinflammation, endocrine control, emotion, learning, and memory (Haas et al., 2008). Wright et al. (2017) found increased levels of HNMT and three types of histaminergic receptors (H1R-H3R) in postmortem dorsolateral prefrontal cortex of ASD patients' brains (Wright et al., 2017). It is noteworthy that Gi-coupled H3R is known to be able to indirectly regulate the other neurotransmitter systems that are involved in ASD pathophysiology owing to the fact that this protein can function as both an autoreceptor and a heteroreceptor. This means that it brings about an inhibition of histamine synthesis and the release of other neurotransmitters, respectively. These properties make H3R a potential therapeutic target for the treatment of related cognitive disorders (Esbenshade et al., 2008; Passani and Blandina, 2011).

### The presence of an abnormal monoamine system in mice with a maternal VPA-induced ASD-like phenotype

Hyperserotonemia is found in ASD patients and an ASD mouse model where the mice had a variant of *SLC6A4* that results in a SERT gain-of-function (Muller et al., 2016). Hyperserotonemia has also been found in VPA-treated animals. Maternal VPA administration not only results in an aberrant migration of serotonin neurons within the embryonic hindbrain, but also their abnormal distribution together with increased tryptophan immunoreactivity in the dorsal raphe nucleus of brains of the adult rats (Miyazaki et al., 2005; Kuwagata et al., 2009; Wang et al., 2013). Serotonin is well known to be involved in melatonin synthesis, and it is also well known that melatonin regulates circadian rhythms; thus the findings in VPA-treated rats of hyperserotonemia of the frontal cortex, hippocampus, cerebellum and blood plasma, when this is linked to the present of abnormal circadian rhythms, implies that there is a disrupted melatonin system in the VPA-treated brain (Narita et al., 2002; Tsujino et al., 2007). Moreover, increased SERT activity is found in the midbrain of *SLC6A4* gain-of-function mice and in the amygdala of VPA-treated mice (Veenstra-VanderWeele et al., 2012; Wang et al., 2013). However, some studies have reported hyperserotonemia in the prefrontal cortex and hippocampus of VPA-treated animals (Dufour-Rainfray et al., 2010; Kumar et al., 2015). Interestingly, reduced levels of serotonin in the intestines of VPA-treated rodents, but increased levels of serotonin in the serum of VPA-treated mice, implies that the presence of defective brain-gut interactions that are associated with ASD pathology (de Theije et al., 2014a,b; Kumar et al., 2015; Lim et al., 2017).

Abnormal midbrain dopamine system has been detected in a VPA-treated ASD model. Dopamine levels and turnover rates in the frontal cortex are higher in VPA-treated rats, which suggests hyperfunctionality of the mesocortical system in these VPA-treated animals (Narita et al., 2002; Nakasato et al., 2008). Dopamine D2 receptor (D2R)-positive pyramidal projection neurons in the mPFC layer V in VPA-treated mice are abnormally activated during social interaction (Brumback et al., 2017). Prenatal VPA exposure reduces methamphetamine-induced increases in extracellular DA levels and also reduces the levels of *Drd1* and *Drd2* mRNAs in the prefrontal cortex; this occurs without alteration in extracellular 5-HT and norepinephrine levels (Hara et al., 2015). In the striatum, alterations in the mesostriatal dopamine pathways remain controversial. Cezar et al. (2018) have reported a reduction in the tyrosine hydroxylase level of the striatum of VPA-treated mice, which suggests hypofunctionality of the mesostriatal dopamine pathways (Cezar et al., 2018). However, Acosta et al. (2018) found that increased levels in the striatum of dopamine and its metabolites are correlated with abnormal timing accuracy and precision when these are measured in an operant lever-press conditioning experiment (Acosta et al., 2018).

Based on the above studies, pharmacological manipulation of the monoamine system has been tested as a strategy for treating ASD. Repression of the 5-HT system by systematic administration of a Gi-coupled 5-HT_1A_ receptor agonist or a Gq-coupled 5-HT_2A_ receptor antagonist has been found to improve ASD-like phenotypes. Chronic treatments with 8-OH-DPAT, a 5-HT_1A_ receptor agonist, not only ameliorates fear memory extinction and social impairment, but also normalizes the E/I balance within the amygdala (Wang et al., 2013). Hara et al. (2017) found that aripiprazole (an atypical antipsychotics that acts as a 5-HT_1A_ receptor agonist and a partial agonist of D2R in order to reduce DA transmission) and risperidone (an atypical antipsychotics that inhibits D2R and the 5-HT_2A_ receptor), but not haloperidol, are able to alleviate reverse recognition memory deficits and social impairment of VPA-treated rats, as well as restoring the reduced number of dendritic spines in the mPFC of VPA-treated rats (Hara et al., 2017). Abnormal vocal communication and striatal compartmentation in VPA-treated mouse neonates are also partially rescued by chronic risperidone treatment after birth (Kuo and Liu, 2017). Notably, risperidone, and aripiprazole are the only two drugs that the FDA has approved for treating irritability among ASD patients (U.S. Food and Drug Administration, 2007, 2009). The pharmacological mechanisms and neurodevelopmental impacts of these pharmacological treatments on the various different brain regions obviously need further investigation.

Administration of melatonin or agomelatine (a dual agonist of melatonin type 1 and type 2 receptors) in VPA-treated rats was found to have beneficial effects, not only on ASD-like phenotypes, but also on the pathophysiology, including improvement in the impairment of CaMKII and PKA signaling in the hippocampus, an increase in blood-brain-barrier (BBB) permeability, a reduction in neuroinflammation, and a lowering of mitochondria dysfunction (Tian et al., 2014; Kumar et al., 2015).

Targeting the catecholamine system has been shown to be an effective way of treating VPA-exposed rodents. In addition to risperidone, which has high affinity to D2R, two other ADHD drugs, atomoxetine and methylphenidate, which act as inhibitors of norepinephrine transporter (NET) and DAT, are able to alleviate ASD-like phenotypes, abnormal catecholamine release, and aberrant dendritic spines in the prefrontal cortex of VPA-treated rats (Choi et al., 2014; Hara et al., 2016). Notably, Hara et al. (2016) found that inhibition of DAT by atomoxetine and methylphenidate has beneficial effects on VPA-induced ASD phenotypes, but not on NET (Hara et al., 2016). These findings are consistent with there being increased extracellular dopamine levels in the prefrontal cortex of VPA-induced ASD animal models after chronic risperidone treatments (Hara et al., 2017).

Notwithstanding the above, there is no evidence as yet to indicate that the histaminergic system in VPA-induced ASD rodents is altered. Nevertheless, administration of the H3R antagonists ciproxifan and 1-(3-(4-tert-pentylphenoxy)propyl)piperidine have been shown to relieve ASD-like behaviors in the VPA-treated mice, as well as reducing oxidative stress and lowering the level of pro-inflammatory cytokines (Baronio et al., 2015; Eissa et al., 2018). These results highlight the therapeutic potential of H3R antagonists as part of the pharmacological treatment of ASD.

## Endocannabinoid signaling

Alterations in neuromodulatory endocannabinoid signaling have been reported in human patients and in ASD animal studies, the latter including VPA-induced rodent models (Chakrabarti et al., 2015; Zamberletti et al., 2017). The presynaptic release of glutamate is able to lead to postsynaptic mGluR-mediated up-regulation of endocannabinoids, including anandamide (AEA), and 2-arachidonoylglycerol (2-AG). The endocannabinoids then pass through the cellular membrane, across the synaptic cleft, which finally leads to retrograde activation of presynaptic cannabinoid receptors; this subsequently induces LTD. This feedback inhibition of synaptic transmission occurs in many brain regions and is involved in the regulation of a diverse range of physiological and pathological functions (Heifets and Castillo, 2009). Alterations affecting the endocannabinoid system have been documented in neurodegenerative and neuropsychiatric diseases related to synaptic plasticity or neuroinflammation; these include Parkinson's diseases, drug addiction, and ASD (Heifets and Castillo, 2009; Mechoulam and Parker, 2013; Chakrabarti et al., 2015; Fernández-Ruiz et al., 2015). Clinical studies support the hypothesis that there is a disruption of endocannabinoid signaling in ASD patients. Down-regulation of cannabinoid receptor type I (CB1R) was first detected in the cerebellum of postmortem ASD patients (Purcell et al., 2001). Up-regulation of cannabinoid receptor type II (CB2R) has been found in the peripheral blood mononuclear cells of ASD children (Siniscalco et al., 2013). Moreover, CB1R gene variants in humans have been linked to the modulation of the striatal response and gaze duration, both in response to social stimulation (Chakrabarti et al., 2006; Chakrabarti and Baron-Cohen, 2011). These findings suggest an involvement of the endocannabinoid system in ASD symptoms. In genetic animal models of ASD, dysfunction of the endocannabinoid system has been found in *Fmr1* and *neuroligin 3* mutant mice (Földy et al., 2013). For example, elevated endocannabinoid-mediated LTD is found in *Fmr1* knockout hippocampus and striatum, and this is correlated with enhanced activity of the 2-AG synthesis enzyme, diacylglycerol lipase (DAGL) (Maccarrone et al., 2010; Zhang and Alger, 2010). Moreover, in these *Fmr1* knockout mice, impaired endocannabinoid-mediated LTD in the striatum and linked behavioral abnormalities are able to be rescued by an inhibition of monoacylglycerol lipase (MGL), an enzyme that degrades 2-AG, (Jung et al., 2012). Inhibition of fatty acid amide hydrolase (FAAH), an AEA hydrolase, is able to reverse the aversive memory and social interaction abnormalities presence in *Fmr1* mutant mice (Qin et al., 2015; Wei et al., 2016). Taken together, the above findings support the use of endocannabinoid modulators as a potential approach to alleviating ASD symptoms in humans (Zamberletti et al., 2017).

Disturbed endocannabinoid signaling seem to contribute to the ASD-like phenotypes in VPA-induced ASD animal models. A reduction in the level of DAGLα in the cerebellum, a lower MGL level in the hippocampus and reduced endocannabinoid signal transduction in the frontal cortex and hippocampus have been found in VPA-exposed rats, despite unaltered levels of CB1R, AEA, and 2-AG (Kerr et al., 2013). Other studies of the brains of VPA-treated rats have found increased phosphorylation of CB1R in the amygdala, hippocampus and dorsal striatum, as well as reduced levels of the AEA synthesis enzyme (Servadio et al., 2016). Furthermore, Kerr et al. (2016) have reported that acute administration of a FAAH inhibitor was sufficient to attenuate social abnormalities, but not the repetitive behavior and defective exploratory behaviors, of VPA-exposed rats with a dimorphic sexual manner (Kerr et al., 2016). On the other hand, Servadio et al. (2016) carried out a modulation of 2-AG degradation by giving an MGL inhibitor and they found there to be an alleviation of social abnormalities, vocalization, repetitive behaviors, and anxiety in the VPA-treated group (Servadio et al., 2016). Deciphering the mechanisms linked to the various distinct elements found within the endocannabinoid system and related to ASD-like phenotypes requires further investigation.

## Dysregulation of canonical WNT signaling and ASD pathophysiology

Wnt signaling is highly conserved and is involved in many neuronal functions including patterning, neurogenesis, axon guidance and synaptogenesis (Rosso and Inestrosa, 2013). Genetic evidence suggests that some molecules that are part of Wnt signaling are also involved in ASD pathogenesis. For example, genetic mutation of the chromodomain helicase DNA binding protein 8 (*CHD8*, a negative regulator of Wnt signaling) and of *CTNNB1* (the β-catenin gene) have been identified as present in ASD patients (O'Roak et al., 2012a,b). ASD has been proposed to involve a developmental disconnection disorder linked to synaptopathy. Several components of Wnt signaling, including the ligands, β-catenin, CHD8, and GSK3β in pre-synaptic and post-synaptic sites, are known to be involved in synaptic development and the regulation of synaptic transmission, both of which affect the E/I balance within the brain (Rosso and Inestrosa, 2013; Caracci et al., 2016). Moreover, Wnt signaling interacts with other signaling pathways that are known to be involved in ASD pathogenesis, such as the mTOR pathway through PTEN (Chen et al., 2015). In addition to synaptic function, Wnt signaling also is known to regulate neuroinflammation in the CNS via NFκB and various other inflammatory pathways (Marchetti and Pluchino, 2013; Ma and Hottiger, 2016). In the Fragile X syndrome genetic mouse model, elevated phosphorylation of GSK3β was found in the *Fmr1* knockout mice, which suggests overactivation of Wnt signaling in this genetic ASD model (Min et al., 2009). Subsequent studies have shown that the ASD-related symptoms of *Fmr1* knockout mice are able to be ameliorated by inhibition of GSK3β (Min et al., 2009; Mines et al., 2010; Guo et al., 2012; Franklin et al., 2014). Based on the versatile roles of Wnt signaling, the Wnt signal pathway has become a prospective target site for the pharmacological treatment of ASD.

Overactivation of Wnt signaling has been reported in rodents that have undergone maternal VPA treatments. Increased phospho-GSK3β and phospho-β-catenin have been found in in the prefrontal cortex, hippocampus, cerebellum and amygdala of these VPA-treated rats (Go et al., 2012; Zhang et al., 2012, 2017b; Qin et al., 2016; Wu et al., 2017b). Moreover, demethylation of the promoter regions, which led to increased mRNA levels of *Wnt1* and *Wnt2*, has been detected in the prefrontal cortex and hippocampus of VPA-treated rats (Wang et al., 2010). Sulindac acts as non-steroidal anti-inflammatory drug (NSAID) and as an anti-metastasis drug and these effect involve the inhibition β-catenin. Prenatal or postnatal administration of sulindac alleviates ASD-like phenotypes in VPA-treated rats, including social abnormalities, repetitive behaviors, hyperactivity, hypersensitivity and learning/memory deficits (Zhang et al., 2012, 2015; Qin et al., 2016). Notably, sulindac treatment not only restores Wnt signaling, but also deactivates mTOR signaling, as well as increasing the number of autophagosomes in VPA-treated brains (Qin et al., 2016). These findings suggest the possibility that the beneficial effects of a Wnt signaling inhibitor may be partially mediated through a restoration of mTOR signaling. It should be noted that an infusion of wortmannin, an inhibitor of the PI3K-AKT-β-catenin pathway, into the amygdala is able to alleviate the social impairment of VPA-treated rats, and that this occurs via a reduction in the over-activation of Wnt signaling (Wu et al., 2017b).

## Neuroinflammation and ASD pathogenesis

Chronic neuroinflammation within the brain has been shown to have deleterious effects and to be associated with neuropsychiatric and neurodegenerative diseases (Najjar et al., 2013; Ransohoff, 2016). Clinical studies have reported evidence that there is neuroinflammation present in ASD brains, including activation of astrocytes and microglia, the presence of elevated levels of cytokines and higher levels of various other inflammatory biomakers (Kern et al., 2015). It is likely that neuroinflammation has a negative effect on ASD pathogenesis, and therefore the intravenous administration of immunoglobulin has been used to suppress the systematic inflammation that may be present in ASD patients (Gupta et al., 1996; Melamed et al., 2018). Behavioral assessments have shown there to be significant improvements in ASD symptoms after intravenous immunoglobulin infusion (Melamed et al., 2018). Maternal immune activation during pregnancy has been reported to significantly increase the probability of psychosis in offspring, both in humans and using animal models (Buka et al., 2001; Shi et al., 2005; Estes and McAllister, 2016; Careaga et al., 2017). Interestingly, two recent studies have shown that social impairment and repetitive behavior induced by maternal immune activation require the presence of segmented filamentous bacteria in the intestine that bring about the release of IL-17a by T_H_17 cells (Choi et al., 2016; Kim et al., 2017b). These studies seem to have uncovered the presence of complex interactions among neural development, the immune system and the gut microbiota. These findings highlight the essential role that the brain-gut axis and inflammation plays in ASD pathogenesis.

Neuroinflammation pathways have been shown to be altered in VPA-induced ASD animal models. Reactive oxygen species (ROS), apoptotic markers, the expression level of NFκB and the levels of pro-inflammatory cytokines are all increased in VPA-treated rodents, whereas the antioxidant glutathione and various anti-inflammatory cytokines are reduced (Tung and Winn, 2011). Moreover, increased BBB permeability and perioxidation, which are indicators of pro-inflammatory responses, have also been found in VPA-exposed animals (Banji et al., 2011; Pragnya et al., 2014; Al-Amin et al., 2015; Gao et al., 2016; Kumar and Sharma, 2016b; Morakotsriwan et al., 2016; Zhang et al., 2017b). Several substances have been tested as methods of reducing this inflammatory response and thus the related ASD-like phenotypes. For example, the antibiotic minocycline reduces not only oxidative stress, nitrosative stress, and BBB permeability in the brain, but also restores gastrointestinal tract motility and reduces excessive inflammation in the ileum (Kumar and Sharma, 2016b). Various natural compounds, including resveratrol, astaxanthin, piperine, docosahexaenoic acid, and palmitoylethanolamide/luteolin, have been shown to have neuroprotective and antioxidative effects using VPA-treated rodent models (Bambini-Junior et al., 2014; Pragnya et al., 2014; Al-Amin et al., 2015; Gao et al., 2016; Fontes-Dutra et al., 2018). Fingolimod and N-acetylcysteine, off-label drugs used to treat multiple sclerosis and acetaminophen overdose, respectively, have been reported to have anti-inflammation and anti-apoptotic effects in the VPA-treated rodent brains (Wu et al., 2017a; Zhang et al., 2017b). Nutrition and food extracts from green tea, Korean red ginseng, purple rice, and silkworm pupae have also been shown to reduce ASD-like phenotypes in VPA-treated rodent models, possibly via an anti-inflammatory mechanism (Banji et al., 2011; Kim et al., 2013; Gonzales et al., 2016; Morakotsriwan et al., 2016; Du et al., 2017). From the above findings, it is clear that further studies are needed to elucidate the various neuro-immune mechanisms underlying VPA-induced pathophysiology. Such mechanisms might include the effects of crosstalk between neurons and glia in the microenvironment of defective brain regions, which in turn might help with the development of treatments for ASD that involve anti-inflammation reagents.

## Epigenetic changes and ASD pathogenesis

Epigenetic changes may occur as part of ASD pathophysiology and these changes often occur as a consequence of genetic alterations and/or environmental stimulation. Previous studies have shown that genes associated with ASD risk often undergo epigenetic modulation during neurodevelopment and these events may be involved in the pathogenesis of ASD (Loke et al., 2015). Genetic mutations of Methyl-CpG binding protein 2 (MECP2), which binds methylated CpG sites and represses gene expression, has been suggested to account for autistic Rett syndrome (Amir et al., 1999). Another example is an increased level of methylation of OXTR, which encodes the oxytocin receptor, which has also been found in ASD patients (Gregory et al., 2009). In response to external environmental stimulation including VPA, increased acetylated levels of histone H3 and H4 have been detected in VPA-treated brains at 6 h after VPA treatments at E12.5 (Kataoka et al., 2011). Moreover, VPA treatment has been shown to increase acetylation in the promoter region of Pax6 in the brain cortex (Kim et al., 2014b). Given that VPA is a potent HDAC inhibitor that is able to regulate gene expression, it was reasonable to test whether ASD-like phenotypes that are induced by maternal VPA treatment are able to be relieved by epigenetic regulators. Treatment with a HDAC inhibitor, including Pentyl-4-yn-VPA, suberoylanilide hydroxamic acid and sodium butyrate, during adulthood not only can relieve ASD-like phenotypes, but also can modulate the acetylation status of neurons in the cerebellum and hippocampus (Foley et al., 2012, 2014; Takuma et al., 2014). In addition to behavioral rescue, sodium butyrate and VPA treatments increase the dendritic spine in hippocampal neurons of prenatal VPA-treated mouse brains (Takuma et al., 2014). These studies suggest that the VPA-induced transient increases in acetylation that occur during the embryonic stages may lead to an abnormal epigenetic state that persists after birth. Genome-wide epigenetic analysis targeting specific regions of VPA-treated brains may thus help to identify the specific genes that are regulated by VPA in the pathogenesis of ASD.

## Conclusion

Here we have reviewed current theories related to ASD pathophysiology and explored the potential treatments available for ASD that have used maternal VPA-induced ASD-like animal models. The findings outlined here should help to provide insights into the pathogenesis of ASD at both a cellular and a molecular level. We have summarized the potential therapeutic candidates identified using VPA-induced animal models of ASD. This type of experimental ASD model has been found to be quite useful clinically because the drugs often prescribed for ASD patients have been found to be able to reduce some ASD-like phenotypes in VPA-treated animals. Thus, we should take advantage of this animal model and use it to screen for new pharmacological compounds that can be used to treat ASD.

ASD is a complex neurodevelopmental disease that includes both genetic and epigenetic components. Abnormalities of the sensory, motor, and limbic systems in the brain are involved in ASD neuropathology. An interesting and important question that remains is how the neural deficits found in broad regions of the brain manifest themselves as ASD core symptoms and allow us to distinguish ASD from other psychiatric diseases. One possibility is that ASD pathology may affect specific cell types within neural circuits across different brain regions. Recent advances in systems neuroscience and single-cell RNA sequencing technology may enable us to address this key issue and help to untangle the complexities of ASD pathophysiology.

## Author contributions

H-YK and F-CL wrote the manuscript.

### Conflict of interest statement

The authors declare that the research was conducted in the absence of any commercial or financial relationships that could be construed as a potential conflict of interest.
